# Usefulness of Four-Point Dermal-Injection Lymphatic Scintigraphy for Lower-Limb Lymphedema

**DOI:** 10.7759/cureus.94941

**Published:** 2025-10-19

**Authors:** Yoshiyuki Kitamura, Takuro Isoda, Sando Motohiro, Masaoki Kusunoki, Hideki Kadota, Shingo Baba, Kousei Ishigami

**Affiliations:** 1 Department of Clinical Radiology, Graduate School of Medical Sciences, Kyushu University, Fukuoka, JPN; 2 Department of Plastic and Reconstructive Surgery, Kyushu University Hospital, Fukuoka, JPN; 3 Department of Health Sciences, Graduate School of Medical Sciences, Kyushu University, Fukuoka, JPN

**Keywords:** four-point dermal-injection lymphatic scintigraphy, lower-limb lymphedema, lymphatic scintigraphy, lymphedema, technetium-99m human serum albumin diethylenetriamine pentaacetic acid

## Abstract

Objective: Lymphatic scintigraphy using radioisotope agents is a good method for evaluating the lymph flow status of patients with lower-limb lymphedema. To depict wide regions of lower-limb lymphatic vessels, at our institution, we administer radioisotopes at four locations in each limb when lymphatic scintigraphy images are to be obtained. Herein, we evaluated the correlation of lymphatic scintigraphy with lymphedema staging and investigated the usefulness of this four-point dermal-injection lymphatic scintigraphy (LyS4).

Materials and methods: The 52 lower limbs of 26 patients (23 females, three males, aged 20-85 years, median 65 years) underwent a LyS4 examination of their lower limbs. The stage of lower-limb lymphedema was diagnosed by plastic surgeons at our institution using the International Society of Lymphology (ISL) staging system. The patients' ISL stages were stage 0 (n=20), stage 1 (n=7), and stage 2 (n=25). No patient was stage 3. For each LyS4 examination, technetium-99m human serum albumin diethylenetriamine pentaacetic acid (99mTc-HSA) was injected. The four injection points were the first interdigital web, slightly below the area of the lateral malleolus, slightly below the area of the medial malleolus, and the lateral side of the feet near the fifth digit of both feet. Using the planar LyS4 images, we investigated the positive/negative appearance of the four lymphatic vessel regions, i.e., anteromedial (AM), anterolateral (AL), posteromedial (PM), and posterolateral (PL) and the dermal backflow (DB). We then examined the correlations of these parameters with the ISL stage.

Results: The absence of AM, the absence of PM, the appearance of DB, and the number of lymphatic vessels depicted were each significant factors for distinguishing lymphedema. The appearance of DB and the number of lymphatic vessels depicted can distinguish moderate lymphedema (ISL stage 0 or 1) from severe lymphedema (ISL stage 2e or 2d). Regarding the diagnostic performance of the LyS4 parameters, high accuracy was observed for the appearance of PM (0.71), that of DB (0.79), and the number of depicted lymphatic vessels (0.77).

Conclusion: LyS4 is a useful method with good correlation with the ISL stage in patients with lower-limb lymphedema.

## Introduction

Lymphedema is a progressive and chronic accumulation of protein-rich lymph fluid in the interstitial space due to a disruption of lymph nodes and lymphatic vessels [1]. Overall, 140-250 million people are estimated to be affected by lymphedema worldwide [1-3]. The diagnosis of lymphedema is difficult, but patients' clinical symptoms and clinical history are of primary importance [1]. The International Society of Lymphology (ISL) proposed the staging system of lymphedema. The ISL system can be used to determine the stage of lymphedema based primarily on the findings of a noninvasive examination when a treatment plan is being considered. This method does not include anatomical features or genetic abnormalities and has room for improvement, but at present it is the only agreed-upon assessment method for pre-treatment evaluation of lymphedema [4].

The use of several imaging modalities to evaluate a patient's lymph flow status has been suggested, including ultrasonography [5], lymphangiography [6,7], lymphatic scintigraphy [8], contrast-enhanced magnetic resonance imaging (contrast-enhanced MR) [9], and indocyanine green (ICG) fluorescence lymphangiography [10]. Among the reports concerning the use of lymphatic scintigraphy for evaluating lower-limb lymphedema, a study by Maegawa et al. classified lymphatic scintigraphy findings in lymphedema cases into five categories; the study also described these findings' relationships with indications for treatment [11]. A correlation between lymphangiography findings and edema staging was reported by Pecking et al. [12]. However, the injection point at radioisotope administration was only the first interdigital web in both the Maegawa et al. and Pecking et al. studies, which depicts a limited region of lymphatic vessels. Our institution also performed lymphoscintigraphy using a single injection previously. This is not a proper verification, but a retrospective review of 56 limbs examined using the single-injection method in past lymphoscintigraphy exams of our institution revealed that lymphatic vessels were detected in nearly all examinations (52/56, 92.9%) in the anterior lower leg region, whereas few cases were observed in the posterior region (6/56, 10.7%).

Shinaoka et al. used ICG fluorescent lymphangiography to depict four lymphatic vessel regions, i.e., anteromedial (AM), anterolateral (AL), posteromedial (PM), and posterolateral (PL) regions. They administered ICG at four locations in each limb and tracked the lymphatic vessels; their analyses revealed that the results correlated with lymphedema staging [13]. Based on that study, we decided to obtain lymphatic scintigraphy images by administering radioisotopes at four locations in each limb. We conducted the present study to evaluate the relationship between lymphatic scintigraphy findings and lymphedema staging and investigate the usefulness of this four-point dermal-injection lymphatic scintigraphy (LyS4) method.

This article was previously presented at the #60 The Japanese Society of Nuclear Medicine, Kyushu Regional Meeting on February 1, 2025.

## Materials and methods

Patients

The patients who underwent a LyS4 examination of their lower limbs at Kyushu University Hospital during the period from March 2024 to March 2025 were included in this study. We excluded patients with a history of surgical treatment of a lower limb. The stage of lower-limb lymphedema was diagnosed by our institution's plastic surgeons, using the ISL staging system [4]. This study was a retrospective study and was conducted in accordance with the tenets of the Declaration of Helsinki and was approved by the institutional review board of Kyushu University (assignment no. 25054).

A final total of 26 patients (52 limbs) were enrolled. The patients were 23 females and three males aged 20-85 years (median 65 years). 

Four-point dermal-injection lymphatic scintigraphy of lower limbs

**Figure 1:**
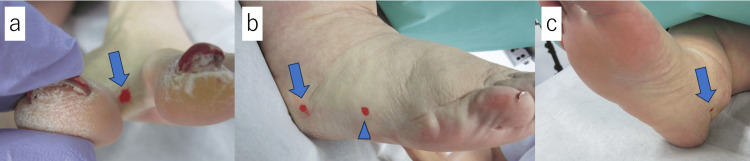
The injection points in the LyS4 examination. a–c: The injection points in the LyS4 examination. Technetium-99m human serum albumin diethylenetriamine pentaacetic acid (^99m^Tc-HSA-DTPA) is injected at the first interdigital web (a: arrow), slightly below the area of the lateral malleolus (b: arrow), the lateral side of the feet near the fifth digit (b: arrowhead) and slightly below the area of the medial malleolus (c: arrow) and of both feet before the image acquisition. LyS4: four-point dermal injection lymphatic scintigraphy

In the patients' LyS4 examinations, technetium-99m human serum albumin diethylenetriamine pentaacetic acid (^99m^Tc-HSA-DTPA) was injected. The four dermal-injection points were the first interdigital web, slightly below the area of the lateral malleolus, slightly below the area of the medial malleolus, and the lateral side of the feet near the fifth digit of both feet (Figure 1), referring to the above-cited Shinaoka et al. study [13]. These injection points were confirmed by a plastic surgeon and marked on the patient's skin before the injection. The injection dose at each point was 70-80 MBq.

A SPECT/CT system (NM/CT 870 CZT, GE HealthCare, Waukesha, WI, USA) and a wide-energy high-resolution (WEHR) collimator were used to acquire whole-body images at 10, 20, 30, 40, 50 and 60 min after the injection of ^99m^Tc-HSA-DTPA. The whole-body imaging protocol consisted of spot images of the whole-body dorsal/ventral view in a matrix of 256×1024 pixels. The whole-body scan was obtained at a 22.0 cm/min table feed rate. SPECT images of each lower limb were obtained with acquisitions in 60 views (10 sec/view) with matrix of 128×128 pixels. CT scans were also performed, with a 5-mm section thickness (130 keV, 30 mAs).

Image evaluation

**Figure 2:**
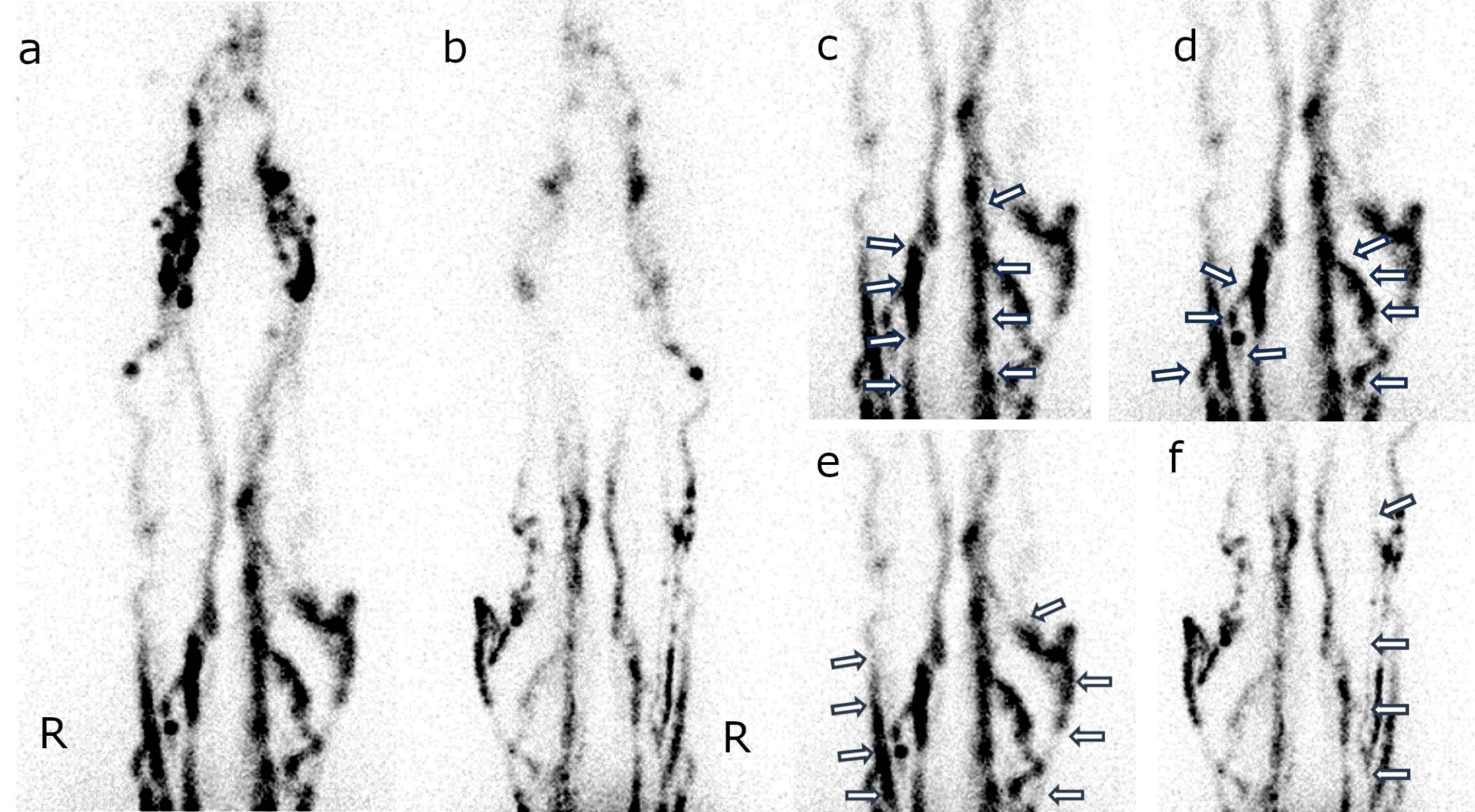
Representative LyS4 images. The anterior (a, c, d, e) and posterior (b, f) planar images 60 min after radioisotope injection. The four lymphatic vessel regions are depicted: posteromedial (PM; c, arrow), anteromedial (AM; d, arrow), anterolateral (AL; e, arrow), posterolateral (PL; f, arrow). The posterolateral lymphatic vessels of the left limb are not depicted in this image. LyS4: four-point dermal injection lymphatic scintigraphy, AL: anterolateral, AM: anteromedial, PL: posterolateral, PM: posteromedial

All whole-body images were referred for image evaluation by two radiologists. As part of the image evaluation, the positive or negative appearance of lymphatic vessels in the four regions (AM, AL, PM, and PL) was determined without considering the degree of accumulation in the lymphatic vessels. If the two radiologists had different judgments, the decision was made by consensus after discussion (Figure 2). Dermal backflow (DB) was considered positive if extra accumulation outside of the lymphatic vessels was observed in the limb. The LyS4 result items, such as the appearance of AM, AL, PM, PL, DB, and the number of the appeared lymphatic vessel regions, were evaluated. The images and patient information used in this study were approved by the IRB as data collected within the observation period. All figures in this manuscript were original data courtesy of our institution.

Statistical analysis

The χ^2^-test was used to determine the correlation between the patients' LyS4 result items and their ISL stages. Probability (p)-values <0.05 were considered significant. All of the statistical analyses were performed using the software program JMP pro 17 (SAS Institute, Cary, NC, USA). Also, the diagnostic performance of the LyS4 for distinguishing lymphedema from a normal limb and for distinguishing moderate lymphedema (ISL stage 0 or 1) from severe lymphedema (ISL stage 2early or 2late).

## Results

The patients' ISL stages of each limb were stage 0 (n=20), stage 1 (n=7), and stage 2 (n=25). The stage 2 cases include 15 early-phase (2early) and 10 late-phase (2late). No patient was stage 3 (Table 1).

**Table 1:** Patient characteristics ISL: International Society of Lymphology

	Male	Female
n (total 26)	3 (11.5%)	23 (88.5%)
Limbs	6 (11.5%)	46 (88.5%)
Age (yrs)	69-72 (median 70)	20–85 (median 60)
ISL Stage		
0	2 (3.8%)	18 (34.6%)
1	4 (7.7%)	3 (5.8%)
2early	0	15 (28.8%)
2late	0	10 (19.2%)
3	0	0

Tables 2, 3 present the correlations between the image evaluation results (i.e., AM, AL, PM, PL, DB, and the number of lymphatic vessels depicted) and the ISL stage. Our analyses revealed that the absence of AM or PM accumulation and the appearance of DB can be used to distinguish lymphedema, with statistical significance. Another significant finding was that two or more lymphatic vessels were depicted if the limb had no symptoms (Table 2). Regarding the differentiation of moderate lymphedema from severe lymphedema, we obtained two significant findings: the patients with severe lymphedema showed the appearance of DB or had 0 or 1 as the number of lymphatic vessels depicted (Table 3).

**Table 2:** The relationship between the ISL stage and the LyS4 image evaluation distinguishing stage 0 from stage 1–2 *χ2-test LyS4: four-point dermal injection lymphatic scintigraphy, ISL: International Society of Lymphology, AL: anterolateral, AM: anteromedial, DB: dermal backflow, n: number of depicted lymphatic vessels, PL: posterolateral, PM: posteromedial. (+)/(-): appeared / not appeared in the LyS4 images.

item		ISL stage		p-value*
		0	1–2	
AM	(+)	16	14	<0.05
	(−)	4	18	
AL	(+)	13	12	0.08
	(−)	7	20	
PM	(+)	12	7	<0.05
	(−)	8	25	
PL	(+)	11	9	0.09
	(−)	9	23	
DB	(+)	3	24	<0.01
	(−)	17	8	
n	0–1	1	21	<0.01
	2–4	19	11	

**Table 3:** The relationship between the ISL stage and the LyS4 image evaluation distinguishing moderate lymphedema (ISL stage 0 or 1) from severe lymphedema (ISL stage 2) *χ2-test LyS4: four-point dermal injection lymphatic scintigraphy, ISL: International Society of Lymphology, AL: anterolateral, AM: anteromedial, DB: dermal backflow, n: number of depicted lymphatic vessels, PL: posterolateral, PM: posteromedial, (+)/(-): appeared / not appeared in the LyS4 images.

item		ISL stage		p-value*
		0–1	2	
AM	(+)	18	12	0.16
	(−)	8	14	
AL	(+)	16	9	0.10
	(−)	10	17	
PM	(+)	13	6	0.08
	(−)	13	20	
PL	(+)	13	7	0.15
	(−)	13	19	
DB	(+)	8	19	<0.01
	(−)	18	7	
n	0–1	5	17	<0.01
	2–4	21	9	

The diagnostic performance of the LyS4 parameters was summarized in Tables 4, 5. Relatively high accuracy for distinguishing lymphedema from a normal limb was observed for the appearance of PM (0.71), DB (0.79), and the number of lymphatic vessels depicted (0.77) (Table 4). Notably, the specificity of the number of lymphatic vessels depicted was high at 0.95. The appearance of DB and the number of lymphatic vessels depicted showed relatively high accuracy for distinguishing moderate lymphedema from severe lymphedema.

**Table 4:** The diagnostic performance of the LyS4 at distinguishing ISL stage 1–2 from stage 0 LyS4: four-point dermal injection lymphatic scintigraphy, ISL: International Society of Lymphology, Acc: accuracy, AL: anterolateral, AM: anteromedial, DB: dermal backflow, n: number of depicted lymph ducts, NPV: negative predictive value, PL: posterolateral, PM: posteromedial, PPV: positive predictive value, Sens: sensitivity, Spec: specificity, (+)/(-): appeared / not appeared in the LyS4 images.

item	Sens	Spec	PPV	NPV	Acc
AM (−)	0.56	0.80	0.82	0.53	0.65
AL (−)	0.63	0.65	0.74	0.52	0.63
PM (−)	0.78	0.60	0.76	0.63	0.71
PL (−)	0.72	0.55	0.72	0.55	0.65
DB (+)	0.75	0.85	0.89	0.68	0.79
n ≤1	0.66	0.95	0.95	0.63	0.77

**Table 5:** The diagnostic performance of the LyS4 at distinguishing moderate lymphedema (ISL stage 0 or 1) from severe lymphedema (ISL stage 2) LyS4: four-point dermal injection lymphatic scintigraphy, ISL: International Society of Lymphology, Acc: accuracy, AL: anterolateral, AM: anteromedial, DB: dermal backflow, n: number of depicted lymph ducts, NPV: negative predictive value, PL: posterolateral, PM: posteromedial, PPV: positive predictive value, Sens: sensitivity, Spec: specificity, (+)/(-): appeared / not appeared in the LyS4 images.

item	Sens	Spec	PPV	NPV	Acc
AM (−)	0.54	0.69	0.64	0.60	0.62
AL (−)	0.65	0.62	0.63	0.64	0.63
PM (−)	0.77	0.50	0.61	0.68	0.63
PL (−)	0.73	0.50	0.59	0.65	0.62
DB (+)	0.73	0.69	0.70	0.72	0.71
n ≤1	0.65	0.81	0.77	0.70	0.73

## Discussion

We described the new lymphatic scintigraphy method, LyS4, determined the correlations of LyS4 parameters with the ISL stage of lower limbs, and evaluated the diagnostic ability of this method. The results of our analyses demonstrated that the appearance of AM, PM, and DB and the number of lymphatic vessels depicted are significant factors that can be used to distinguish ISL stage 0 from ISL stage 1. In addition, the appearance of DB and the number of lymphatic vessels depicted are significant factors for distinguishing ISL stages 0 and 1 from ISL stage 2. Regarding the diagnostic performance of LyS4, the accuracy values distinguishing ISL stage 0 from ISL stages 1 and 2 were as follows: the appearance of DB (0.79) and the number of lymphatic vessels depicted (0.77); the corresponding accuracy values for distinguishing ISL stages 0 and 1 from ISL stage 2 were 0.71 and 0.73, respectively. In these results, the absence of PM visualization may indicate the presence of stage 1 or higher lymphedema. Furthermore, the number of lymphatic vessels specifically visualized by LyS4 is useful for distinguishing the presence or absence of lymphedema and may also provide information regarding its severity. The PM visualization and the number of lymphatic vessels are specific findings obtained by the LyS4, which is less likely to be obtained by the single-injection methods, highlighting the potential utility of LyS4.

In the Maegawa et al. study of ICG fluorescence lymphangiography, the accuracy of the appearance of DB for distinguishing ISL stage 0 from ISL stage ≥1 was 0.80, and the accuracy for distinguishing ISL stages 0 and 1 from ISL stage ≥2 was 0.78 [11]. These data are similar to those obtained in the present study.

Using the original staging system as a reference for scintigraphy results, Pecking et al. reported that the accuracy for distinguishing ISL stage 0 from ISL stage ≥1 was 0.88, and the accuracy for distinguishing ISL stages 0 and 1 from ISL stage ≥2 was 0.96 [12]. Those results are very good, but that study involved a single-point injection examination. In addition, the speed of lymphatic transportation, which may depend on individual decisions, should be taken into account.

**Figure 3:**
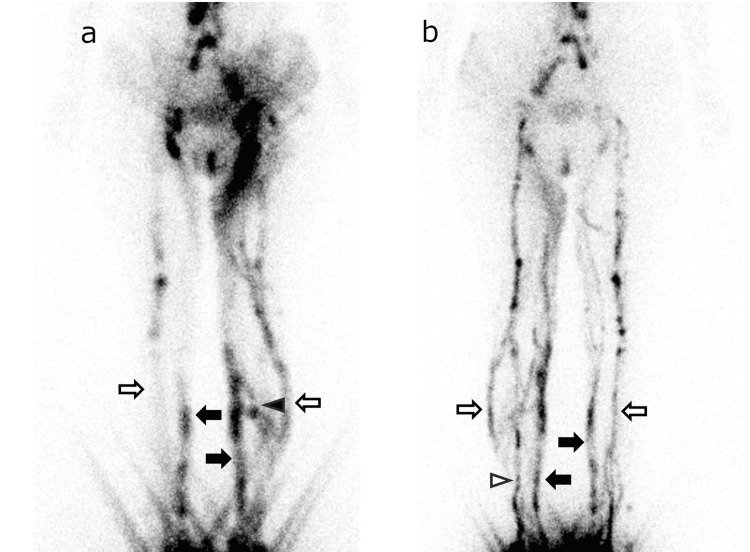
Anterior and posterior LyS4 images of a female in her 30s with ISL stage 1 lymphedema of the left limb. Anterior (a) and posterior (b) LyS4 images of a female in her 30s with ISL stage 1 lymphedema of the left limb. These images were acquired 60 min after injection. All four lymphatic vessels appeared with DB at the proximal thigh in the left limb. The right limb, which was diagnosed as normal, showed PM and AL, but AM and PL were not detected (white arrow: AL, black arrow: PM, white arrow head: AM, black arrow head: PL) LyS4: four-point dermal injection lymphatic scintigraphy, ISL: International Society of Lymphology, AL: anterolateral, AM: anteromedial, DB: dermal backflow, PL: posterolateral, PM: posteromedial

**Figure 4:**
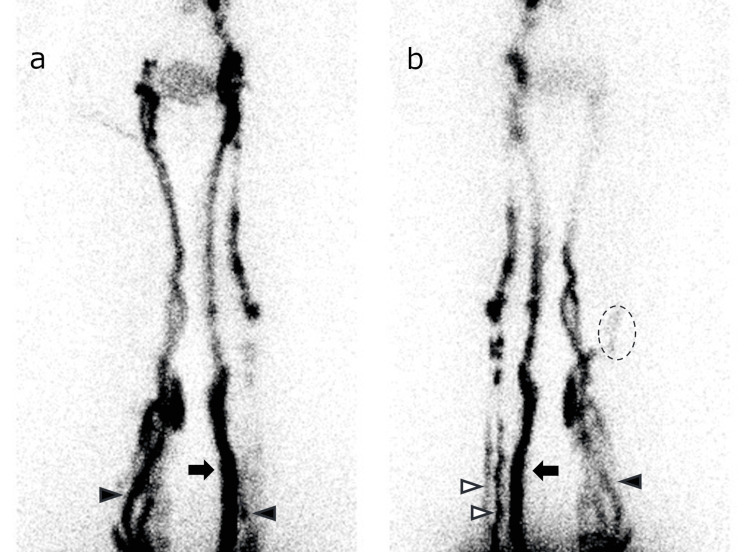
Anterior and posterior LyS4 images of a female in her 40s with the late-phase of ISL stage 2 lymphedema of the right limb. Anterior (a) and posterior (b) LyS4 images of a female in her 40s with the late-phase of ISL stage 2 lymphedema of the right limb. These images were acquired 60 min after injection. Only AM appeared in the right limb with DB at the distal lower leg. In the left limb, which was diagnosed as the normal limb, AM, PM and AL were detected with no DB (white arrow: AL, black arrow: PM, white arrow head: AM, black arrow head: PL, dotted circle: DB). LyS4: four-point dermal injection lymphatic scintigraphy, ISL: International Society of Lymphology, AL: anterolateral, AM: anteromedial, DB: dermal backflow, PL: posterolateral, PM: posteromedial

The LyS4 method can delineate lymphatic vessels in all areas of the lower limbs, especially deeper lymphatic vessels that are relatively difficult to delineate with ICG fluorescence lymphangiography (Figures 3, 4). Detailed lymphatic flow information is expected to provide additional information that will be more useful in determining treatment strategies for lymphedema, the indications for lymphatic venous anastomosis, and the selection of the surgical site.

Our present findings demonstrate that LyS4 provides more information about lymphatic vessels than conventional single-point lymphatic scintigraphy while maintaining adequate evaluation performance. Several types of examination are available for evaluating lymphatic vessels in lymphedema of the lower extremities (Table 6). The LyS4 examination is superior to ultrasonography and ICG fluorescence lymphangiography for revealing details of lymphatic vessels. It is also superior to lymphangiography in its relative simplicity. At the point of radiation exposure, contrast-enhanced lymphatic MR produces no radiation exposure; however, it is difficult to perform in patients for whom MR is contraindicated or who are allergic to MR contrast media. These patients can undergo LyS4 with no limitation. Therefore, although there is a slight radiation exposure, LyS4 is unique in that it provides objectively good lymphatic vessel delineation.

**Table 6:** Various examinations for lower-extremity lymphedema ICG: indocyanine green fluorescence lymphangiography, LA: lymphangiography, LyS: lymphatic scintigraphy, LyS4: four-point dermal injection lymphatic scintigraphy, MR: magnetic resonance imaging, US: ultrasonography.

Method	LyS4	Ordinal LyS [8]	US [5]	MR [9]	LA [6,7]	ICG [10]
Complexity	Simple	Simple	Difficult	Simple	Difficult	Simple
Depiction of lymphatic vessels	Good	Limited	Limited	Good	Good	Limited
Invasiveness	Slight	Slight	None	Slight	High	Slight
Radiation exposure	Expose	Expose	None	None	Expose	None
Other limitation				Be aware of the risk of an allergy to contrast media. Patients with MR contraindications cannot be tested.	Be aware of the risk of an allergy to contrast media.	

We did not use any parameters from SPECT/CT images as a criterion for evaluation in this study. This is because the imaging range of SPECT/CT was not standardized in the patient population, making it difficult to create evaluation parameters. We agree that SPECT/CT is useful for the evaluation of lymphatic vessels as Pecking et al. reported [12]. An evaluation method that incorporates SPECT/CT imaging is a topic for a future study. We also did not address treatment decision-making or the prognostic value of LyS4 findings. We believe that the LyS4 method is useful for determining treatment strategies and predicting treatment efficacy, but further case collections and follow-ups are needed to verify this.

The proximal lymphatic system in the pelvis, abdomen, and chest was not mentioned in this study. In this study, the appearance of the lymph nodes was weaker than lymphatic vessels in almost all cases. We think that this is because we take images limited to 60 minutes after injection, which results to make weak uptake. Although not shown in the images presented in this report, visualization of the cisterna lactea, thoracic duct, and left venous angle was good, and images comparable to those obtained with the one-point method appear to have been acquired. 

There are several limitations of this study. First, the number of patients in this study was small. We believe the LyS4 is a suitable method for evaluating lymphoedema; however, the number of cases is limited due to its novelty. Second, this study is a retrospective design; therefore, a prospective or multicenter evaluation would be beneficial. Third, the ISL stage 3 cases were not observed. This is because our institution rarely encounters severely progressed lymphoedema patients. We think the usefulness of LyS4 can be argued based on its potential to aid in treatment decision-making during early edema stages, even without including the ISL stage 3 case. However, this issue could potentially be resolved through multicenter research. The third point is that SPECT images of LyS4 were not used as diagnostic parameters in this study. Parameters with SPECT images may be useful for diagnosis with anatomical information compared to planar images; however, the timing of the SPECT examination is controversial. Furthermore, as noted in the Introduction, lymphoscintigraphy using a single injection provides poor visualization of posterior lymphatic vessels, making LyS4 potentially more useful in this regard. However, no reports have verified this point and necessitating precise validation.

## Conclusions

For the evaluation of lower-limb lymphedema, the LyS4 method, i.e., four-point dermal-injection lymphatic scintigraphy, is a relatively simple method that provides clear images of lymphatic vessels. To confirm the severity of lower-limb lymphedema, the number of lymphatic vessels depicted and the depiction of dermal backflow by LyS4 may be one of the viewpoints. LyS4 is expected to contribute to decisions about the treatment strategies for lymphedema of the lower extremities and assessments of the efficacy of treatment.
